# Fasting before Intra-Gastric Dosing with Antigen Improves Intestinal Humoral Responses in Syrian Hamsters

**DOI:** 10.3390/vaccines12060572

**Published:** 2024-05-24

**Authors:** Liam Wood, Jaime Hughes, Mark Trussell, Anne L. Bishop, Ruth Griffin

**Affiliations:** 1 Vaccines and Therapeutics Group, School of Life Sciences, The University of Nottingham Biodiscovery Institute, University Park, Nottingham NG7 2RD, UK; 2 Clostridia Research Group, Synthetic Biology Research Centre (SBRC), The University of Nottingham Biodiscovery Institute, University Park, Nottingham NG7 2RD, UK; 3 School of Life Sciences, The University of Nottingham Biodiscovery Institute, University Park, Nottingham NG7 2RD, UK; 4 Bio Support Unit, The University of Nottingham Medical School, Nottingham NG7 2UH, UK; 5 Parasites and Microbes Programme, Wellcome Sanger Institute, Wellcome Genome Campus, Hinxton, Cambridge CB10 1SA, UK; 6 NIHR Nottingham Biomedical Research Centre, Nottingham University Hospitals NHS Trust, The University of Nottingham, Nottingham NG7 2UH, UK

**Keywords:** oral vaccines, *Clostridioides difficile*, intestinal delivery, fasting, secretory IgA

## Abstract

**Simple Summary:**

Vaccines prevent approximately 4 million deaths annually worldwide but around 3 times this number still die each year from infectious diseases. Most vaccines are injected, generating immune responses in the bloodstream but injected vaccines may be less effective against gut bugs that cause localised infections such as *Clostridioides difficile*. Oral vaccines have the advantage of promoting immune responses within the small intestine, protecting the gut. To develop an oral vaccine against *C. difficile*, the conventional animal model is the hamster as it develops similar clinical symptoms to humans. A challenge for oral vaccines is overcoming the harsh conditions of the stomach; however, capsules can be used that resist stomach acidity and dissolve once in the small intestine. We previously tested a protein vaccine candidate for responses in the small intestine following oral administration to hamsters using capsules. As the responses were variable, we questioned whether delivery could be improved after fasting hamsters. Using the same protein, we compared intestinal responses in fasted versus fed groups. The fasted group demonstrated significantly greater responses suggesting improved delivery of the capsules to the intestine. Our refined immunisation method can now be applied by the growing number of groups worldwide using the hamster model to test oral vaccines.

**Abstract:**

Oral vaccines, unlike injected, induce intestinal secretory immunoglobulin A (sIgA) mimicking our natural defense against gut pathogens. We previously observed sIgA responses after administering the *Clostridioides difficile* colonisation factor CD0873 orally in enteric capsules to hamsters. Enteric-coated capsules are designed to resist dissolution in the stomach and disintegrate only at the higher pH of the small intestine. However, the variable responses between animals led us to speculate suboptimal transit of antigens to the small intestine. The rate of gastric emptying is a controlling factor in the passage of oral drugs for subsequent availability in the small intestine for absorption. Whilst in humans, food delays gastric emptying, in rats, capsules can empty quicker from fed stomachs than from fasted. To test in hamsters if fasting improves the delivery of antigens to the small intestine, as inferred from the immune responses generated, 24 animals were dosed intragastrically with enteric capsules containing recombinant CD0873. Twelve hamsters were fasted for 12 h prior to each dose and the other 12 fed. Significantly higher sIgA titres, with significantly greater bacterial-adherence-blocking activity, were detected in small intestinal lavages in the fasted group. We conclude that fasting in hamsters improves intestinal delivery leading to more robust responses.

## 1. Introduction

Vaccination is the most cost-effective medical intervention for disease prevention; however, communicable diseases collectively remain the second leading cause of death worldwide [1]. There is an urgent need for new and improved vaccines that can directly target the initial infection site, which, for 90% of infections, are mucosal sites [2]. To this end, immunising via the oral route is attracting interest, as not only does it induce protective responses in the intestinal tract, but also in distal mucosal sites and systemically [3]. Furthermore, without the need for needles, oral vaccines are safer and cheaper and can be self-administered. In order to develop new vaccines, suitable animal models that can represent the characteristics of infection in humans are required. 

For over 60 years, the Syrian hamster (*Mesocricetus auratus*) has been used to study over 70 different viral infections and at least six parasitic or bacterial diseases [4]. Several studies have reported that Syrian hamsters are more representative of humans than murine models for the analysis of viral infections with regard to disease symptoms, pathogenesis and immune responses [4,5,6,7]. Specifically, it has been demonstrated that human cytokines, including granulocyte-macrophage colony-stimulating factor (GM-CSF) and interleukin-12 (IL-12), are fully functional in hamster models, but not in mouse models [8,9]. More recently, the Syrian hamster has become the model of choice for studying SARS-CoV-2 infection [10,11,12]. SARS-CoV-2, like SARS-CoV, uses the cellular surface protein angiotensin-converting enzyme 2 (ACE2) to bind and enter cells. Unlike murine ACE2, which does not effectively bind the viral spike protein, hamster ACE2 is homologous to that of humans [13] and binds well, making hamsters susceptible to infection. Upon experimental intra-nasal infection with SARS-CoV-2, Syrian hamsters show robust viral replication and moderate to severe clinical signs very early after infection, including weight loss and pulmonary pathology similar to that of humans [13]. Studies investigating the performance of oral and nasal vaccines directed against SARS-CoV-2 in the Syrian hamster have shown promising immunogenicity profiles [14] and protection from lung pathology, pulmonary viral load and clinical disease following viral challenge [14,15,16,17,18,19]. 

Since the 1970s, the Syrian hamster has been a widely used model for the study of *Clostridioides difficile* infection (CDI) [20,21]. The hamster is profoundly more susceptible to CDI than mice and develops many of the clinical symptoms observed in humans [22,23,24]. As such, this lethality model has been invaluable for the testing of vaccines and therapeutics [25,26,27,28,29,30,31,32,33]. *C. difficile* is a Gram-positive, spore-forming anaerobe that resides in the intestinal tract of humans and animals and in the environment. The life cycle of the bacillus begins with the ingestion of faecal orally transmitted spores. The spores resist the proteolytic enzymes and acidity in the stomach and enter the small intestine where they germinate into vegetative cells [34]. Antibiotic-induced gut dysbiosis enables this opportunistic pathogen to colonise the colon where cells multiply and secrete toxins, which destroy the actin cytoskeleton of enterocytes and disrupt the tight junctions [35,36]. Symptoms range from mild diarrhoea to pseudomembranous colitis, which may be accompanied by toxic megacolon [37,38]. In hamsters, challenge with a toxigenic strain of *C. difficile*, following antibiotic administration, results in haemorrhagic caecitis. Fulminant disease symptoms include “wet tail” (diarrhoea), ruffled fur, hunching and lethargy, with death ensuing usually within 48 h post-inoculation [20,21,28,39].

With the failure of two intra-muscular toxoid vaccines to prevent primary CDI in Phase III trials [40,41], attention is turning to oral approaches. Encouragingly, we have observed in the hamster model partial protection from CDI following oral immunisation with recombinant *C. difficile* colonisation factor CD0873 [28]. Importantly, CD0873 is safe as shown by histological analysis of the small intestine of orally immunised hamsters [29]. One of the challenges for oral vaccines is the need to overcome the harsh conditions of the stomach, in particular proteases and acidity. In humans, the stomach pH is 2–3, while the small intestine and colon are pH 6.5–7 and pH 7–7.8, respectively [42]. In pharmacotherapy, drugs are generally taken orally to be absorbed systemically from the small intestine, which has a very large surface area (100 m^2^); thus, there has been considerable interest in the enteric coating of pharmaceutical preparations [43,44]. These include drugs that cause gastric irritation (e.g., non-steroidal anti-inflammatory drugs), drugs that produce nausea or drugs that are acid-labile in gastrointestinal (GI) fluid such as enzymes or peptides [45]. Adopting oral delivery approaches originally developed for drugs such as gastro-resistant capsules has enabled progress in the development of oral vaccines such as Vivotif against *Salmonella enterica* serovar Typhi [46].

The passage of the dosage form along the gastro-intestinal tract can influence the absorption of a drug [47]. Gastric emptying, the process by which the contents of the stomach are moved into the duodenum for absorption in the small intestine, can be a factor contributing to the onset of absorption [48]. In humans, food delays gastric emptying and subsequent evacuation of capsules to the small intestine [45]. However, a study in rats found that capsules emptied quicker from fed stomachs than from fasted [49]. Like most oral drugs, the target site for oral vaccines is the small intestine, specifically the ileum, being the main immune inductive site. 

The goal of this study was to test if fasting improves the passage of antigens to the small intestine in Syrian hamsters. Using our established intestinal immunogen, CD0873 was administered in enteric-coated gelatin capsules to fed and fasted groups and humoral responses in intestinal lavages compared.

## 2. Materials and Methods

### 2.1. Bacterial Strains and Culture Conditions

*Clostridioides difficile* strain 630 was obtained from ATCC (Manassas, VA, USA) and cultured in Brain Heart Infusion (BHI) broth or on BHI agar (Oxoid, Basingstoke, UK) supplemented with 0.5% yeast extract (Oxoid, Basingstoke, UK) and 0.1% cysteine (ThermoFisher Scientific, Loughborough, UK) (BHIS), and with 250 µg/mL D-cycloserine and 8 µg/mL cefoxitin (Oxoid, Basingstoke, UK) to make BHIS CC. The strains were incubated overnight at 37 °C in an anaerobic workstation (Don Whitley Scientific, Bingley, UK) with an atmosphere of CO_2_ (10%), H_2_ (10%) and N_2_ (80%). Media were pre-reduced before use.

### 2.2. Assessment of pH of the Gastrointestinal (GI) Tract of Hamsters

The pH of the GI tract contents of six 12-week-old female Golden Syrian hamsters (Janvier Labs, Le Genest-Saint-Isle, France) was measured using a hand-held pH/mV/C meter c/w (HI-1230B) and a pH electrode with a micro bulb and body (H1-1083B) (Hanna Instruments Ltd., Bedfordshire, UK). The hamsters were euthanised by CO_2_ overdose and death confirmed by cervical dislocation. pH readings were taken immediately afterwards at multiple points along the GI tract in triplicate.

### 2.3. Preparation of Capsules

Recombinant CD0873 was produced as described previously [28,29]. One mL aliquots of 1 mg/mL protein in PBS were mixed with 1 mg of cryoprotectant, Trehalose, lyophilised and then packed into size 9 gelatin capsules (Torpac) using the manufacturer’s stand, funnel and tamper as before [28,29]. Capsules were dip-coated once using the polymer mixture of 12.5% EUDRAGIT L100 (Evonik Industries, Essen, Germany) in isopropanol, with 10% (*w*/*w*) plasticiser, Triethyl citrate (TEC) and 3% (*v*/*v*) H_2_O [28,29].

### 2.4. Protein Stability Assays

To test the stability of recombinant CD0873 after lyophilisation, a CD0873-loaded capsule (2.3) was emptied in 1 mL PBS. Once the lyophilised protein was reconstituted as determined by the appearance of a homogenous suspension, 100 µL were removed and added to 100 µL of 2× sample buffer. Ten or 5 µL were fractionated, along with the non-lyophilised form, by SDS-PAGE (12% acrylamide) for Coomassie staining and Western immunoblotting, respectively, as previously described [27]. 

To test the stability of recombinant CD0873 in small intestinal fluid, the small intestines of 6 freshly euthanised 8-week-old female Golden Syrian hamsters (Janvier Labs) were gently flushed with 1 mL PBS, intestinal contents were centrifuged for 10 min and supernatants were combined, re-centrifuged and then filter-sterilised. The contents of 7 CD0873-loaded capsules (2.3) were emptied into 7 separate Eppendorf tubes and 375 µL of intestinal fluid were added. The mixture was agitated until the lyophilised protein had resolubilised. The Eppendorfs were incubated at 37 °C with gentle shaking for 2 min, 5 min, 10 min, 15 min, 30 min, 1 h or 2 h, and then 100 uL were removed and added to 100 uL of 2× sample buffer and frozen. Samples were analysed by SDS-PAGE (12% acrylamide) fractionation, Coomassie staining and Western analysis as described previously [27]. 

### 2.5. In Vivo Study

Golden Syrian female hamsters aged 12 weeks (Janvier Labs) were housed in pairs in individually ventilated cages. Hamsters were randomly divided into 2 groups, each with *n* = 12. After acclimatisation for 1 week, hamsters were intra-gastrically dosed with a single capsule using the dosing syringe provided by the capsule manufacturer (Torpac) on days 1, 15 and 30. Both groups were given constant free access to drinking water (tap water that has undergone filtering through 5 µm, carbon and 0.5 µm filters). One group was fasted for 12 h (during the dark cycle when active and normally feeding) before dosing while the other group was fed. Hamsters were euthanised 14 days after the final immunisation. Small intestines were collected and individually placed immediately in 1 mL ice-cold PBS containing SIGMAFAST™ protease inhibitors (Sigma, Merck Group, Feltham, UK), then flushed with a further 3 mL. Lavages were transferred to 50 mL falcon tubes and centrifuged at maximum speed for 10 min at 4 °C. Supernatants were collected and centrifuged again until clear, filter-sterilised and then stored in aliquots at −80 °C. 

### 2.6. Direct ELISA for Detection of Total sIgA in Intestinal Fluid

Hamster sIgA ELISA kits were purchased from MyBioscource, San Diego, CA, USA. Plates, reagents and samples were brought to room temperature prior to use. Fifty μL of standards supplied with the kit or 50 μL neat intestinal fluid were added to the plate in triplicate. HRP-conjugated reagent (100 μL) was added to each well and the plates were incubated at 37 °C for 1 h. The wells were washed 5 times with 200 μL PBST (PBS, 0.01% (*v*/*v*) Tween-20). Fifty μL of chromogen reagent was added to each well and the plates were incubated at 37 °C for 15 min. Stop solution was added and the optical density was read within 5 min at *A*_450nm_. Antibody titres were determined from the standard curve generated. The medians of technical triplicates within one assay and the means for two independent assays were calculated for each animal sample.

### 2.7. Adherence Blocking Assay

Caco-2 cells (ATCC, HTB-37) were maintained in Corning^®^ 25 cm^2^ cell culture flasks (Corning, NY, USA) in 15 mL Dulbecco’s Modified Eagles Medium (DMEM) (Gibco™ ThermoFisher Scientific, Loughborough, UK) containing 10% (*v*/*v*) fetal bovine serum (FBS) and 1% (*w*/*v*) penicillin/streptomycin (Sigma). Cells were incubated at 37 °C with 5% CO_2_. Cells were seeded at a density of 1 × 10^5^/mL in a total volume of 500 μL per well in a 24-well plate (Corning^®^), then grown for 14 days with the media changed every 2–3 days. Caco-2 cells were counted using a haemocytometer and a Motic AE2000 light microscope. For the week leading up to the assay, cells were maintained in serum-free DMEM containing 1% (*w*/*v*) penicillin/streptomycin. The media were changed again 24 h before conducting the assay.

A 10 mL overnight broth culture of *C. difficile* was grown and 1 mL was centrifuged at 3500× *g* for 2 min, washed in PBS and centrifuged again. The pellet was re-suspended in serum-free DMEM and an appropriate dilution was used as inoculum to achieve the desired multiplicity of infection (MoI). For MoI verification, 100 μL of the bacterial cell suspension was serially diluted in PBS (10^−1^ to 10^−11^) and 100 μL of each dilution plated onto BHIS CC. 

The entirety of the adherence assay was performed under anaerobic conditions at 37 °C. Fifty μL of intestinal fluid (diluted 1:2 BHIS) were added to 50 μL of inoculum and incubated for 1 h. The mixture was then added to Caco-2 cells with 400 μL serum-free DMEM, following the removal of spent DMEM, and incubated for 2 h. Non-adherent bacteria were removed by aspirating, and then the adherent bacteria were harvested as follows. Caco-2 cells were washed 2 times with 500 μL PBS and then incubated with 200 μL 1× trypsin-EDTA (Sigma) for 10 min to detach them from the wells. The detached cells were re-suspended in 300 μL DMEM with 10% FBS. The cell suspension was serially diluted (10^−1^ to 10^−3^) in BHIS and 10 μL of each dilution was spotted in triplicate on BHIS CC plates and incubated overnight. CFUs were enumerated the following day and expressed as CFU per 100 Caco-2 cells.

### 2.8. IgG Indirect ELISA for Detection of Antigen-Specific IgG in Serum

Nunc 96-well MaxiSorp^™^ plates were coated with 100 µL of purified recombinant CD0873 [27,28,29] at a concentration of 0.5 µg/mL in 0.2 M sodium bicarbonate coating buffer at pH 9.4 and the proteins were left to adsorb overnight at 4 °C. All wash stages consisted of 5 washes with 200 µL PBST. First, the wells were blocked with 200 μL of 5% (*w*/*v*) skimmed milk powder (Sigma, Merck Group, Feltham, UK) in PBST for 2 h at room temperature, washed and then incubated overnight at 4 °C with 100 μL serum diluted 1:75 in PBST in triplicate. Wells were washed and incubated for 2 h at room temperature with 100 μL goat anti-hamster IgG (H + L) highly cross adsorbed-Biotin antibody (Sigma) at a 1:20,000 dilution in PBST. Wells were washed again and incubated at room temperature with 100 μL Streptavidin-HRP (R&D Systems, Minneapolis, MN, USA) diluted at a ratio of 1:200 in PBST for 1 h followed by a wash. Plates were incubated with 100 μL of TMB (Sigma Merck Group, Feltham, UK)) for 15 min at room temperature and the reaction stopped by the addition of 100 μL 2M H_2_SO_4_. *A*_450nm_ was read on a CLARIOstarPlus (BMD LABTECH, Ortenberg, Germany) plate reader. 

For the standards, IgG generated by Life Science Group Ltd., Bedfordshire, UK (formerly Antibody Production Services, UK) [27], was used. The antibody stock of 4.08 mg/mL was diluted in PBS to 1:10^6^ and 1:20, 1:30. 1:40, 1:50, 1:60, 1:70 and 1:80 dilutions of this stock were plated. For these wells, the IgG-HRP antibody (#7074, Cell Signaling Technology, Danvers, MA, USA) was used at a 1:5000 dilution in PBST.

### 2.9. Ethics Statement

Animal studies were devised using the Experimental Design Assistant (EDA) online tool and conducted in strict accordance with the requirements of the Animals Scientific Procedure Act 1986. Prior approval for these procedures was granted by the University of Nottingham Animal Welfare and Ethical Review Body and by the United Kingdom Home Office and the work conducted under project license PP2643068. Animals were euthanised by CO_2_ inhalation to minimise suffering and death was confirmed by cervical dislocation.

### 2.10. Statistical Analysis 

Data were checked for normality with the Shapiro test and equal variance with Levene’s test before proceeding with parametric tests. Normally distributed data were analysed by unpaired 2-tailed T-tests. Data that were non-normally distributed were analysed by a Mann–Whitney U test. Statistical tests were performed using GraphPad Prism version 10 and R version 4.3.2. 

## 3. Results

The Syrian hamster is a widely used model for investigating viral and bacterial infections and the efficacy of vaccines directed against these pathogens. In recent years, it has become the main model for studying SARS-CoV-2 [10,11,12] and is the longstanding model for the study of *C. difficile* [22,23,24] and corresponding vaccines and therapeutics [25,26,27,28,29,30,31,32,33]. Oral immunisation against viral and bacterial pathogens is attracting interest since deploying this route of administration can elicit intestinal sIgA and systemic IgG, mimicking our natural first and second lines of defense, respectively, unlike injected vaccines, which mainly induce serum IgG. Oral immunisation is particularly important for non-invasive enteric pathogens such as *C. difficile.* Indeed, injected toxoid vaccines have failed to demonstrate protection from primary CDI in Phase III trials likely due to the ineffective induction of local, intestinal responses [40,41]. 

We previously observed strong intestinal responses after immunising hamsters orally with capsules containing CD0873 which had been lyophilized in cryoprotectant [28,29], with one in four hamsters clearing infection after subsequent challenge with a hypervirulent strain of *C. difficile* [28]. However, the results were variable between individual hamsters of the same group. This variation could be due to inherent differences between hamsters in their stomach pH or small intestine pH, which may affect capsule dissolution and cargo release. Alternatively, limitations of the cargo itself may affect biological outcomes, including integrity following lyophilisation and room temperature storage and stability in intestinal fluid. Another factor likely to cause variation is the efficiency of transit of cargo to the small intestine. It is known that food delays gastric emptying and if capsules are retained for long enough in the stomach, they become permeable to the gastric milieu, which dissolves the capsule and this, in turn, disrupts the polymer coat resulting in capsule disintegration, dispersal of contents and degradation [45]. 

### 3.1. CD0873 Is Stable Following Lyophilisation and Storage and Further Incubation in Intestinal Fluid

**Figure 1:**
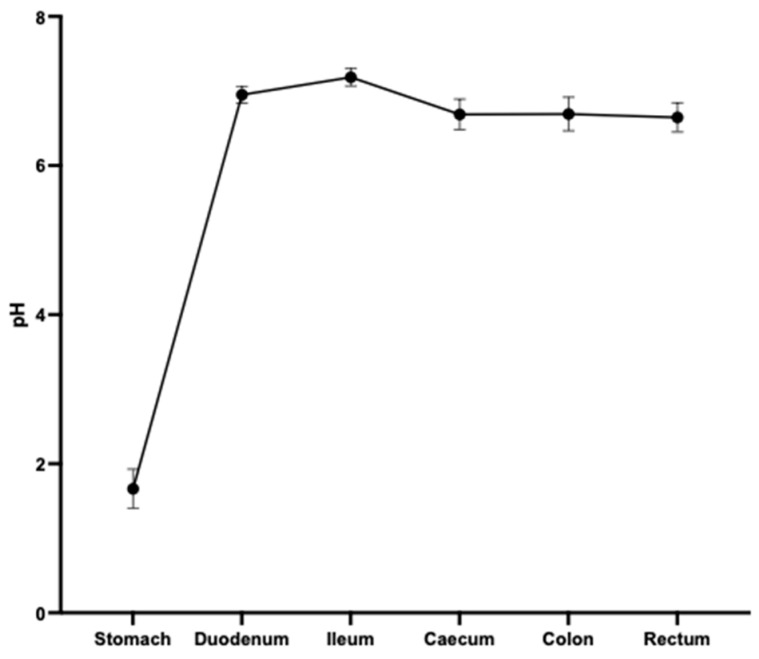
The pH along the gastrointestinal tract of 6 Golden Syrian hamsters with triplicate readings taken from each region of each animal. The averages of the means of the triplicates are shown with standard deviations displayed by bars.

First, to test whether there is variability between hamsters in the pH of their stomachs and small intestines, six hamsters were euthanised humanely and pH readings immediately taken of the stomach, duodenum and ileum, along with the large intestine. No profound differences were observed between the hamsters for any region tested (Figure 1). 

**Figure 2:**
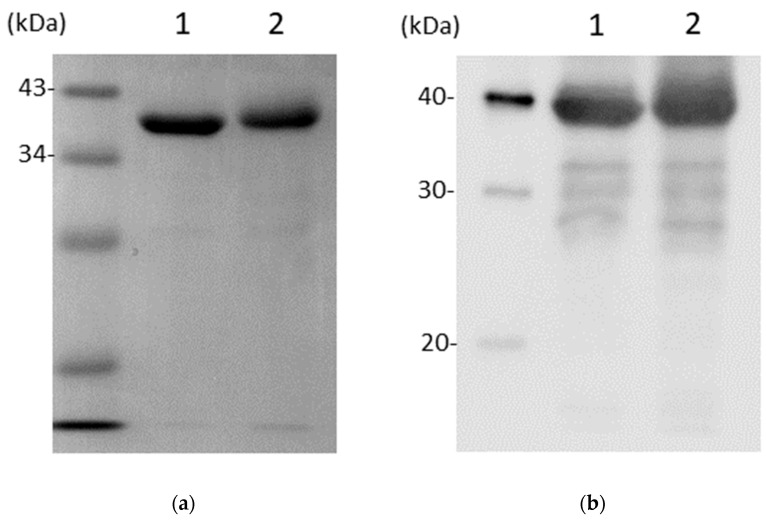
Stability of antigen CD0873 following lyophilisation and packing in a gelatin capsule and long-term storage at room temperature. The integrity of the lyophilised protein was compared with protein from the same batch that had been frozen. Ten or 5 µL of 1 mg/mL of each protein was fractionated by SDS-PAGE (12% acrylamide) and stained with Coomassie Blue (**a**) or immunoblotted with rabbit anti-CD0873 antibody (**b**), respectively. Bands were detected by the addition of anti-rabbit horseradish peroxidase-conjugated IgG secondary antibody and ECL Western Blotting Detection Reagent.

To test the stability of the recombinant protein following lyophilisation and room temperature storage, the contents of a CD0873-loaded capsule, prepared for the fast study 20 months previously, were reconstituted in PBS and assessed for degradation by SDS-PAGE and Western immunoblotting with the rabbit anti-CD0873 antibody we used previously [27]. Fractionation patterns were compared with protein from the same batch that had not been lyophilised, and instead stored at −80 °C. Protein from the two differently stored preparations showed equal preservation with only very minor degradation: 3 faint bands were detected by Western immunoblotting (Figure 2b) but were barely detectable by Coomassie staining (Figure 2a). We conclude CD0873 is highly stable following lyophilisation and long-term storage at room temperature. 

**Figure 3:**
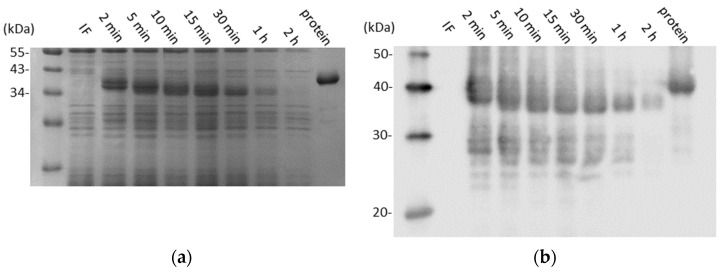
Stability of recombinant CD0873 in hamster intestinal fluid (IF) over time, determined by SDS-PAGE fractionation (12% acrylamide) and staining by Coomassie Blue (**a**) and Western immunoblotting with anti-CD0873 antibody (**b**). Control samples include IF alone and CD0873 protein alone.

Next, to test the stability of CD0873 in the small intestinal milieu, seven hamsters were euthanised and the fluid from the small intestine harvested and pooled. The contents of seven more capsules previously prepared for the fasting study were individually incubated in the pooled fluid at 37 °C with gentle shaking for different lengths of time to mimic host conditions. Prior to incubation, the lyophilised antigen was fully re-solubilised in the intestinal fluid by agitation. SDS-PAGE fractionation and Western immunoblotting demonstrated reasonable stability of the antigen (Figure 3). The action of intestinal proteases was evident from 2 min post-incubation, with progressive reduction in the size of the protein up to 10 min. After this, the protein remained the same size and could still be detected by Coomassie up to 1 h (Figure 3a). Western immunoblotting with the anti-CD0873 antibody corroborated observations made by Coomassie staining with additional faint detection of the antigen at 2 h (Figure 3b)

### 3.2. Mucosal Antibody Responses to CD0873 Given Intra-Gastrically in Capsules Are Significantly Greater in Fasted versus Fed Hamsters

To test if fasting improves the passage of antigen to the small intestine, intra-gastric administration of enteric capsules containing 1 mg of lyophilised CD0873 was conducted as before [28,29]. Hamsters were dosed with a single capsule 3 times, 2 weeks apart, and then euthanised 2 weeks later. One group (*n* = 12) was fasted for 12 h before each dose and another group was fed (*n* = 12). Both groups were given free access to drinking water at all times. 

Intestinal lavages were tested for total sIgA titres by sandwich ELISA and for antibody functionality in an adherence blocking assay using the Caco-2 human intestinal epithelial cell line, an effective *C. difficile* in vitro adherence model described by Kovacs-Simon et al. (2014) [50]. Three independent experiments were conducted using different multiplicities of infections (MoI) within the original range (MoI: 5 and 100) used [50]). *C. difficile* cells were pre-incubated with intestinal lavages for 1 h before adding to Caco-2 cells. Loosely bound bacteria were removed and Caco-2 cells were detached from wells with trypsin. Serial dilutions of the cell suspensions were plated to enumerate bacteria. 

**Figure 4:**
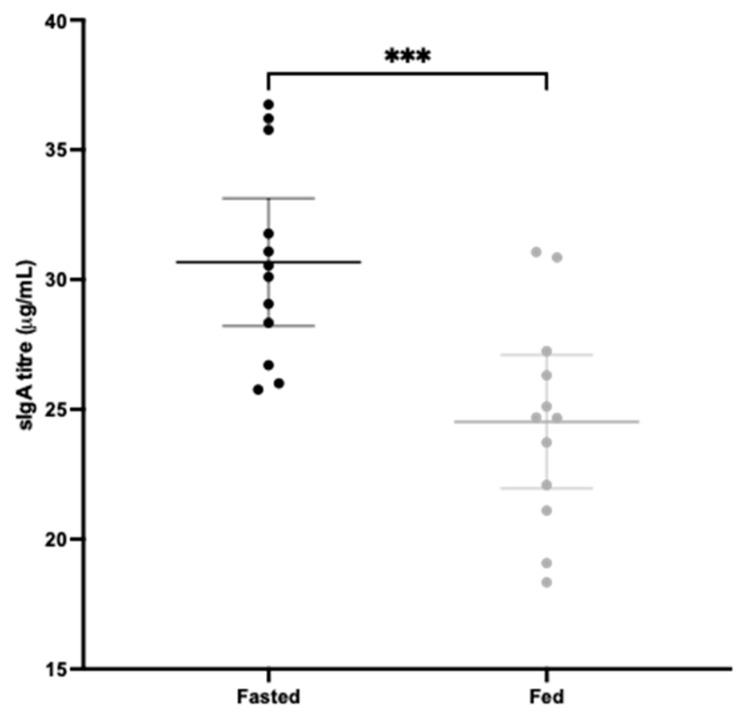
Total sIgA in small intestinal lavages of fasted and fed hamsters measured by a total sIgA ELISA kit. Lavages were taken at the experimental endpoint, 2 weeks after intra-gastric administration of 3 capsules containing CD0873. The means of 2 independent ELISA assays and medians of technical triplicates within each assay are shown. Middle bars represent the mean and outer bars represent 95% confidence intervals. The data were analysed by an unpaired 2-tailed T-test. Statistical difference *p* value: *** *p* ≤ 0.001.

**Figure 5:**
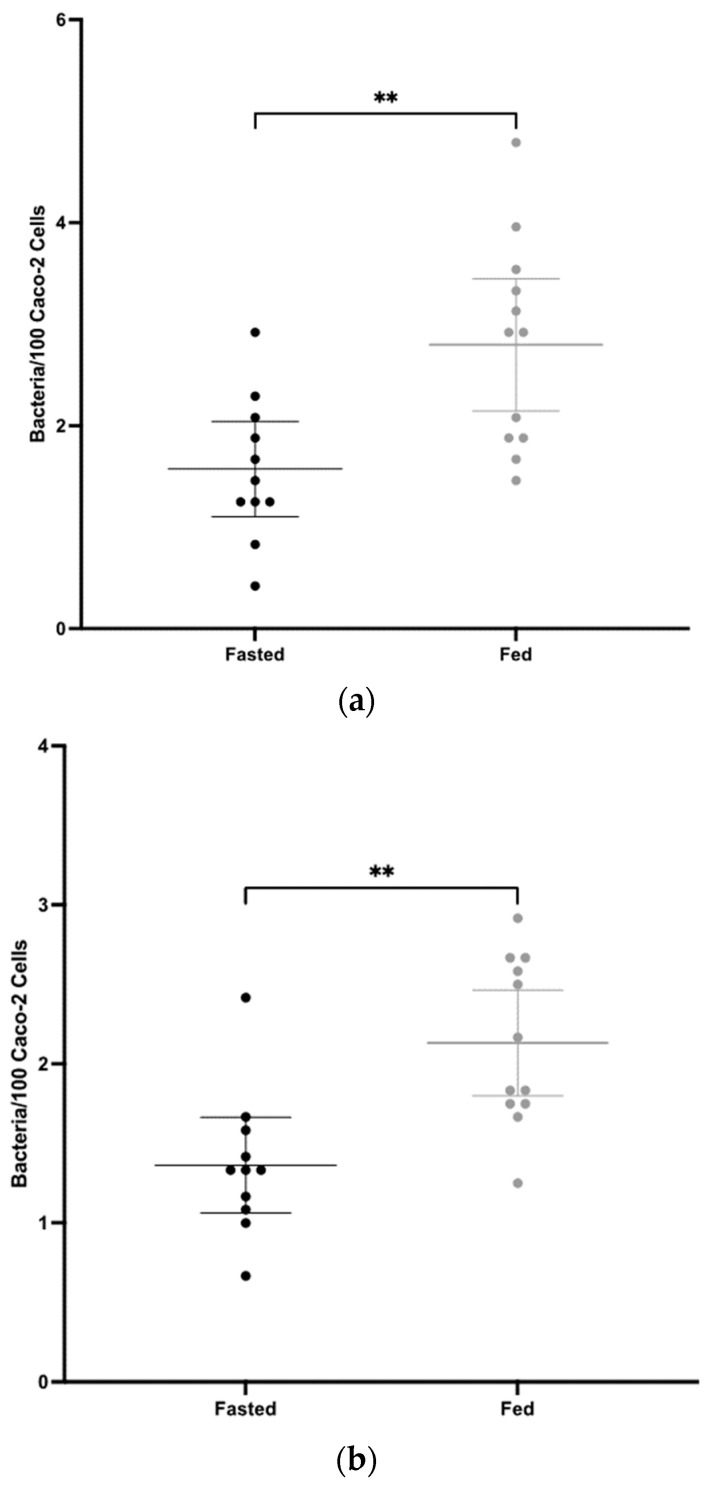

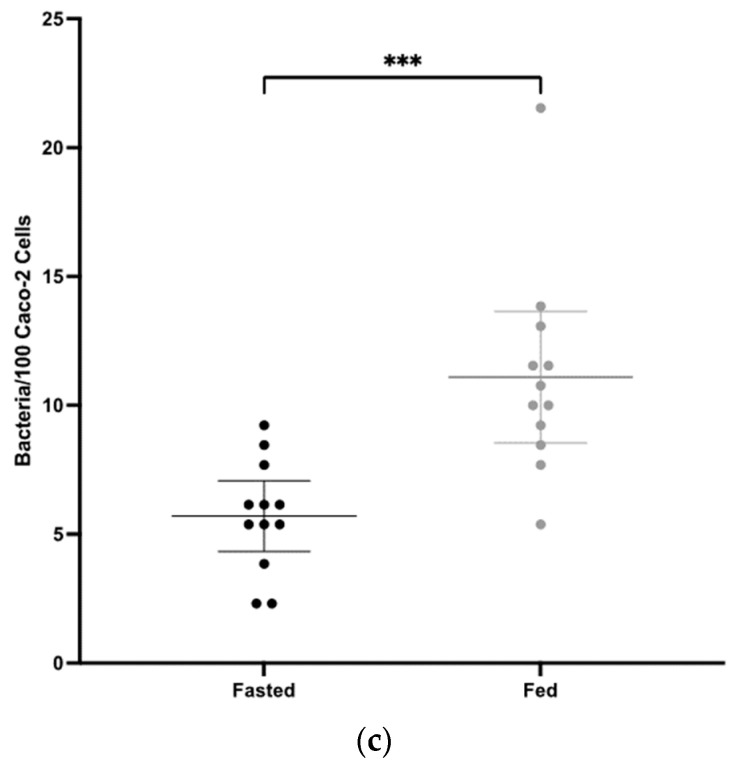
Adherence of cells of *C. difficile* strain 630 to Caco-2 monolayers following their pre-incubation in intestinal lavages of fasted and fed hamsters, diluted 1:2. Three different sized inocula of *C. difficile* were used; MoI 20 (**a**), MoI 25 (**b**) and MoI 80 (**c**). Adherence was measured by enumerating CFU from washed, trypsinised Caco-2 cells. Each dot represents one animal. Middle bars represent the mean and outer bars represent 95% confidence interval. The data were analysed by an unpaired 2-tailed T-test. Statistical difference *p* value: *** *p* < 0.001, ** *p* < 0.01.

The fasted group showed a significantly greater mean sIgA titre in intestinal lavages than the fed group, where *p* = 0.0010 (Figure 4). In all three adherence assays, significantly greater mean blocking activity was observed in the fasted group compared to the fed group. As the MoI increased, significance increased; for MoI 20, *p* = 0.0033, for MoI 25, *p* = 0.0011 and for MoI 80, *p* = 0.0005 (Figure 5). The highly significant result for the last experiment using a heavy inoculum demonstrates strong functionality of the antibodies generated. There was little visible difference in the degree of variation between the two groups for the sIgA titre (Figure 4) or for adherence blocking activity (Figure 5). Levene’s test confirmed no significant difference in variance.

### 3.3. Systemic Antibody Responses to CD0873 Given Intra-Gastrically in Capsules Are Greater in Fasted versus Fed Hamsters

**Figure 6:**
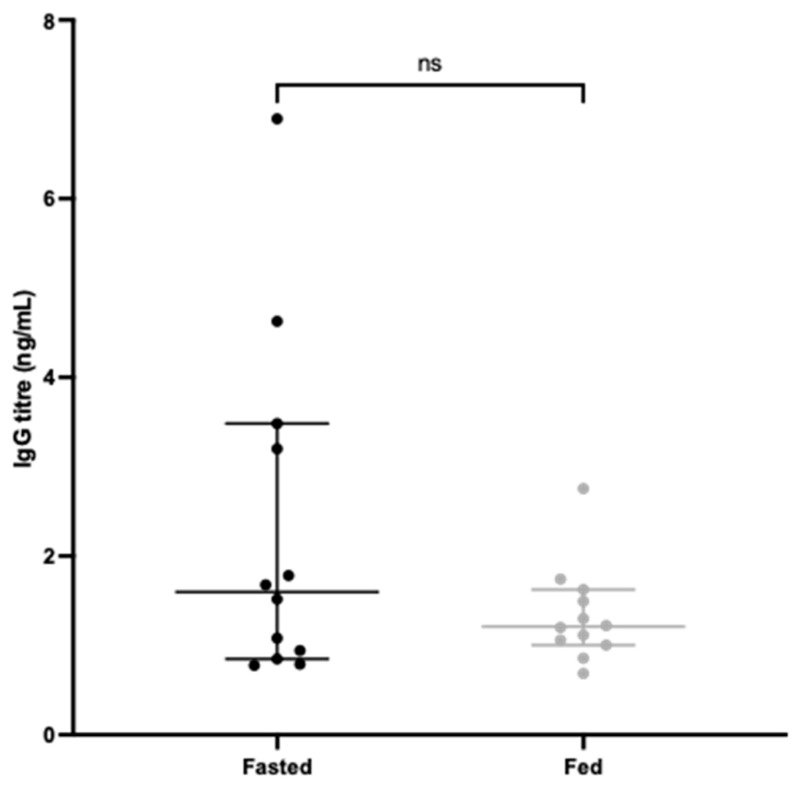
Systemic antibody responses in fasted and fed hamsters dosed intra-gastrically with capsules containing CD0873. Sera were diluted 1:75 in PBST and quantified for CD0873-specific IgG by indirect ELISA. Goat anti-hamster IgG highly cross-adsorbed Biotin antibody (1:20,000) and Streptavidin-HRP (1:200) were used for detection. Middle bars represent the mean and outer bars represent 95% confidence interval. The data were analysed by a Mann–Whitney U test and found to be non-significant.

To test if fasting also influences the systemic IgG response, blood was harvested by cardiac puncture at the experimental endpoint and serum tested in an indirect ELISA to quantify CD0873-specific IgG for each animal. Figure 6 shows that the fasted group exhibited higher IgG titres than the non-fasted group, although the overall difference was non-significant.

**Figure 7:**
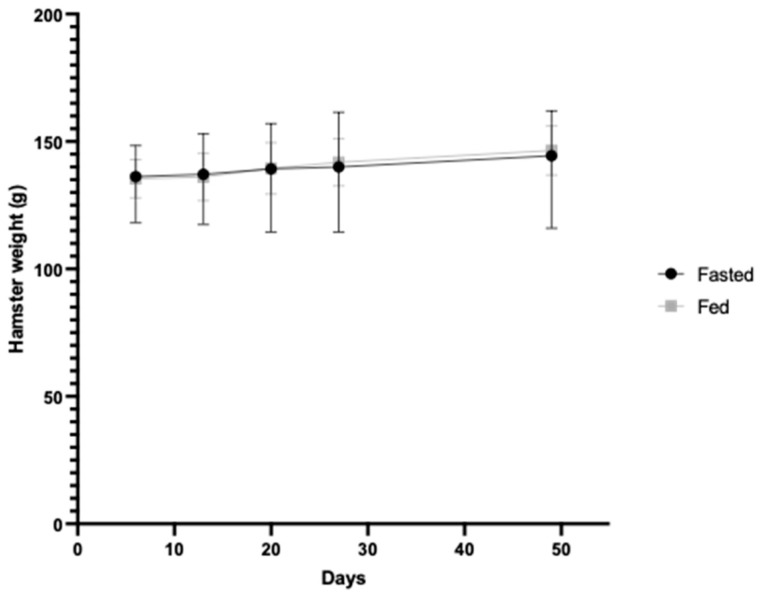
Mean weight (grams) of fasted and fed hamsters from day 6 (one day before the first dose) to the end of the study. The black and grey bars represent the range of weights of the animals in fasted and fed groups.

Finally, since the Torpac capsules used in this study were designed for rodents over 150 g and very few hamsters reached this weight, we assessed whether dosing was more effective in heavier hamsters. No effect of weight on immunological responses per animal was observed. The effect of fasting on weight per se was also assessed. Both groups showed similar mean weights over the time course of the study (Figure 7). 

## 4. Discussion

The goal of this study was to test if a 12 h fast in hamsters improves the transit of antigens to the small intestine as inferred from elevated humoral responses. The *C. difficile* colonisation factor, CD0873, that we previously found to be a potent intestinal immunogen in the hamster model [27,28,29] was used for this study. 

Firstly, we tested the stability of the antigen: (1) following lyophilisation and 20 months storage at room temperature and (2) following subsequent incubation in hamster intestinal fluid with gentle shaking at body temperature to mimic the intestinal environment. For the first test, upon reconstitution in PBS, the lyophilised antigen showed similar integrity to the protein from the same batch that had been stored at −80 °C. For the second test, the antigen showed reasonable stability in intestinal fluid. After some initial protease action, the protein could still be detected up to 1 h by Coomassie and up to 2 h by Western immunoblotting. In this test, the protein was fully re-solubilised in intestinal fluid prior to incubation at 37 °C. However, in vivo, we anticipate the protein remaining in its lyophilised form for longer, delaying protease attack and degradation until naturally re-solubilised.

For lyophilised protein to reach the small intestine in the first place, we deployed gelatin capsules coated in enteric polymer based on the work of Staelen et al. (2016). In this study, a multimodal imaging approach was used to longitudinally monitor the GI transit of capsules in Syrian hamsters [51]. Gelatin capsules were filled with contrast agents, ^19^ F-FDG and BaSO_4_, and dip-coated in a L100 EUDRAGIT polymer mixture. Imaging was conducted by ^19^F magnetic resonance imaging (MRI) and computed tomography (CT). Importantly, ^19^F-MRI visualised the capsule becoming water permeable 2 h after oral administration and CT monitoring showed the capsule reached the small intestine of one hamster but remained trapped in the stomach of the other three hamsters after 6 h. 

We previously tested reducing the thickness of the polymer mixture to achieve a more even coating by adding 3% (*v*/*v*) water [28]. Four hamsters were orally administered a single capsule containing BaSO_4_ and capsule location, integrity and dispersal of BaSO_4_ were longitudinally monitored by CT. We observed intact capsules at 1.5 h, and their entry into the small intestine at 3 h at which point, half the capsule was shown to be disintegrated [28]. However, like Staelens, we too have observed prolonged capsule retention in the stomach of some hamsters with no subsequent detection in the small intestine suggesting disintegration in the stomach. Indeed, the variable humoral responses we observed between hamsters dosed orally with antigen-loaded capsules [28,29] corroborate the variable efficiency of capsule transit and subsequent cargo release.

For formulation to reach the small intestine, capsule transit depends on gastric emptying. The rate of gastric emptying is a carefully regulated process consisting of different phases and is accomplished by the following three mechanisms: (1) peristaltic waves, (2) systolic contractions of the antrum, and (3) reduction in size of the stomach [52]. It is generally thought that the presence of food delays gastric emptying leading to prolonged retention of capsules in the stomach. Hunter et al. (1980) longitudinally monitored disintegration of non-coated hard gelatin capsules given orally to three human subjects both in fasted and non-fasted states [53]. For one subject, the presence of food dramatically slowed down gastric emptying, and for all subjects, there was little dispersal of the capsule contents in the stomach in the fasted state, holding promise for greater availability in the small intestine [53]. A study by Kenyon et al. (1994) further investigated the effect of food on the behaviour of enteric coated capsules in humans (although starch capsules were used instead of gelatin) and support the notion that feeding causes a delay in the transit of capsules [45]. However, a study in rats demonstrated the opposite finding. Saphier et al. investigated the GI transit of enterically coated hard gelatin capsules by X-ray imaging and found that more rats retained the capsules in the stomach in the fasted state than in the fed state 2.5 h after dosing [49]. 

Using the polymer mixture we previously optimised [51], and recombinant CD0873 that we found to be an effective intestinal immunogen [28,29], we were able to test whether a 12 h fast prior to intra-gastric dosing in hamsters improves intestinal delivery of antigen. Both the mean sIgA and mean adherence blocking activity of intestinal lavages of fasted hamsters were significantly greater than those of fed hamsters, suggesting improved passage of capsules to the small intestine (Figure 4 and Figure 5). The similar degree of variation between the two groups however suggests further improvement in intestinal targeting is needed. Increasing the number of capsules administered could increase the chance of more capsules reaching the small intestine. Additionally, shorter length capsules such as size 9 h (5.1 mm) instead of size 9 (8.4 mm) (Torpac) may empty more efficiently from the stomach. Indeed, Saphier et al. observed in rats that enteric-coated gelatin capsules from Capsugel, 7.18 mm in length, did not readily evacuate from the stomach during the period of monitoring (up to 12 h after dosing), but reducing their length to 4.8 mm or 3.5 mm resulted in efficient emptying within 3 h [49]. 

It is difficult to interpret the different outcomes in hamsters in our study and rats in the study by Saphier et al. [49]; however, it should be noted that in our study, hamsters were fasted during the night when normally active and feeding, and dosed early morning, whereas the rats were fasted during the day when usually sleeping, and dosed in the evening. Differences in the capsule used may have also affected outcomes, including brand, a different enteric polymer and dip-coating six times in the rat study [49] instead of once in our study.

Aside from intestinal targeting, another bottleneck to overcome in the development of oral vaccines is the need for effective delivery systems to enable the transcytosis of antigens to the gut associated lymphoid tissue (GALT). Antigens are thought to be primarily transcytosed to Peyers patches via microfold cells (M cells) and over many decades, research has been conducted on identifying M cell receptors to improve targeting [54]. Another limitation for oral vaccines is identifying suitable adjuvants to evoke sufficiently strong responses, and to date, only Cholera Toxin subunit B has been approved, which is included in a killed whole cell oral vaccine against cholera (Dukoral) [55]. A novel delivery system that can carry cargo antigens and adjuvant to the GALT to induce local protective responses would be a game changer for the development of new oral vaccines. Aside from their mucosal immunological benefits over injected vaccines, their practicality (being needle-free) from a cost, administration and safety perspective makes oral vaccines extremely desirable for mass immunisation and pandemic preparedness [3].

## 5. Conclusions

To summarise, from this study, we conclusively demonstrate that fasting increases small intestinal humoral responses, significantly raising the concentration of sIgA with significantly increased capacity to block the adherence of *C. difficile* cells in a gut epithelial model. Additionally, fasting resulted in an increase in antigen-specific systemic IgG. The improved responses are likely to result in improved protection from CDI and this warrants further testing in a challenge study. We conclude that fasting is an important step to include in study protocols testing orally or intra-gastrically administered formulations to improve delivery to the small intestine in the widely used Syrian hamster model. 

## Data Availability

Data are contained within the article.

## References

[1] Vela Ramirez J.E., Sharpe L.A., Peppas N.A. (2017). Current state and challenges in developing oral vaccines. Adv. Drug Deliv. Rev..

[2] Hoft D.F., Brusic V., Sakala I.G. (2011). Optimizing vaccine development. Cell Microbiol..

[3] Kwong K.W.-Y., Xin Y., Lai N.C.-Y., Sung J.C.-C., Wu K.-C., Hamied Y.K., Sze E.T.-P., Lam D.M.-K. (2023). Oral Vaccines: A Better Future of Immunization. Vaccines.

[4] Miao J., Chard L.S., Wang Z., Wang Y. (2019). Syrian Hamster as an Animal Model for the Study on Infectious Diseases. Front. Immunol..

[5] Ogg M., Jonsson C.B., Camp J.V., Hooper J.W. (2013). Ribavirin protects Syrian hamsters against lethal hantavirus pulmonary syndrome–after intranasal exposure to Andes virus. Viruses.

[6] Prescott J., DeBuysscher B.L., Brown K.S., Feldmann H. (2014). Long-term single-dose efficacy of a vesicular stomatitis virus-based Andes virus vaccine in Syrian hamsters. Viruses.

[7] Zu Rhein G.M. (1983). Studies of JC virus-induced nervous system tumors in the Syrian hamster: A review. Prog. Clin. Biol. Res..

[8] Cho S.-A., Park J.-H., Seok S.-H., Juhn J.-H., Kim S.-J., Ji H.-J., Choo Y.-S., Park J.-H. (2006). Effect of granulocyte macrophage-colony stimulating factor (GM-CSF) on 5-FU-induced ulcerative mucositis in hamster buccal pouches. Exp. Toxicol. Pathol..

[9] Wang P., Li X., Wang J., Gao D., Li Y., Li H., Chu Y., Zhang Z., Liu H., Jiang G. (2017). Re-designing Interleukin-12 to enhance its safety and potential as an anti-tumor immunotherapeutic agent. Nat. Commun..

[10] Lei Y., Zhang J., Schiavon C.R., He M., Chen L., Shen H., Zhang Y., Yin Q., Cho Y., Andrade L. (2021). SARS-CoV-2 Spike Protein Impairs Endothelial Function via Downregulation of ACE 2. Circ. Res..

[11] Yadav P.D., Mohandas S., Shete A.M., Nyayanit D.A., Gupta N., Patil D.Y., Sapkal G.N., Potdar V., Kadam M., Kumar A. (2022). SARS-CoV-2 Kappa Variant Shows Pathogenicity in a Syrian Hamster Model. Vector-Borne Zoonotic Dis..

[12] Yuan S., Ye Z.-W., Liang R., Tang K., Zhang A.J., Lu G., Ong C.P., Poon V.K.M., Chan C.C.-S., Mok B.W.-Y. (2022). Pathogenicity, transmissibility, and fitness of SARS-CoV-2 Omicron in Syrian hamsters. Science.

[13] Imai M., Iwatsuki-Horimoto K., Hatta M., Loeber S., Halfmann P.J., Nakajima N., Watanabe T., Ujie M., Takahashi K., Ito M. (2020). Syrian hamsters as a small animal model for SARS-CoV-2 infection and countermeasure development. Proc. Natl. Acad. Sci. USA.

[14] Chau E.C.T., Kwong T.C., Pang C.K., Chan L.T., Chan A.M.L., Yao X., Tam J.S.L., Chan S.W., Leung G.P.H., Tai W.C.S. (2023). A Novel Probiotic-Based Oral Vaccine against SARS-CoV-2 Omicron Variant B.1.1.529. Int. J. Mol. Sci..

[15] Bellier B., Saura A., Luján L.A., Molina C.R., Luján H.D., Klatzmann D. (2022). A Thermostable Oral SARS-CoV-2 Vaccine Induces Mucosal and Protective Immunity. Front. Immunol..

[16] Jawalagatti V., Kirthika P., Hewawaduge C., Yang M.-S., Park J.-Y., Oh B., Lee J.H. (2022). Bacteria-enabled oral delivery of a replicon-based mRNA vaccine candidate protects against ancestral and delta variant SARS-CoV-2. Mol. Ther..

[17] Johnson S., Martinez C.I., Tedjakusuma S.N., Peinovich N., Dora E.G., Birch S.M., Kajon A.E., Werts A.D., Tucker S.N. (2022). Oral Vaccination Protects Against Severe Acute Respiratory Syndrome Coronavirus 2 in a Syrian Hamster Challenge Model. J. Infect. Dis..

[18] Lloren K.K.S., Jawalagatti V., Hewawaduge C., Chandran S., Park J.-Y., Lee J.H. (2023). Salmonella-mediated oral delivery of multiple-target vaccine constructs with conserved and variable regions of SARS-CoV-2 protect against the Delta and Omicron variants in hamster. Microbes Infect..

[19] Wang S., Cui H., Zhang C., Li W., Wang W., He W., Feng N., Zhao Y., Wang T., Tang X. (2023). Oral delivery of a chitosan adjuvanted COVID-19 vaccine provides long-lasting and broad-spectrum protection against SARS-CoV-2 variants of concern in golden hamsters. Antivir. Res..

[20] Bartlett J.G., Onderdonk A.B., Cisneros R.L., Kasper D.L. (1977). Clindamycin-associated colitis due to a toxin-producing species of *Clostridium* in hamsters. J. Infect. Dis..

[21] Price A.B., E Larson H., Crow J. (1979). Morphology of experimental antibiotic-associated enterocolitis in the hamster: A model for human pseudomembranous colitis and antibiotic-associated diarrhoea. Gut.

[22] Borriello S.P., Barclay F.E. (1985). Protection of hamsters against *Clostridium difficile* ileocaecitis by prior colonisation with non-pathogenic strains. J. Med. Microbiol..

[23] Buckley A.M., Spencer J., Candlish D., Irvine J.J., Douce G.R. (2011). Infection of hamsters with the UK *Clostridium difficile* ribotype 027 outbreak strain R20291. J. Med. Microbiol..

[24] Razaq N., Sambol S., Nagaro K., Zukowski W., Cheknis A., Johnson S., Gerding D.N. (2007). Infection of hamsters with historical and epidemic BI types of *Clostridium difficile*. J. Infect. Dis..

[25] Bruxelle J.F., Tsapis N., Hoys S., Collignon A., Janoir C., Fattal E., Péchiné S. (2018). Protection against *Clostridium difficile* infection in a hamster model by oral vaccination using flagellin FliC-loaded pectin beads. Vaccine.

[26] Huang J.-H., Wu C.-W., Lien S.-P., Leng C.-H., Hsiao K.-N., Liu S.-J., Chen H.-W., Siu L.-K., Chong P. (2015). Recombinant lipoprotein-based vaccine candidates against *C. difficile* infections. J. Biomed. Sci..

[27] Hughes J., Aston C., Kelly M.L., Griffin R. (2022). Towards Development of a Non-Toxigenic *Clostridioides difficile* Oral Spore Vaccine against Toxigenic *C. difficile*. Pharmaceutics.

[28] Karyal C., Hughes J., Kelly M.L., Luckett J.C., Kaye P.V., Cockayne A., Minton N.P., Griffin R. (2021). Colonisation Factor CD0873, an Attractive Oral Vaccine Candidate against *Clostridioides difficile*. Microorganisms.

[29] Karyal C., Palazi P., Hughes J., Griffiths R.C., Persaud R.R., Tighe P.J., Mitchell N.J., Griffin R. (2021). Mimicking Native Display of CD0873 on Liposomes Augments Its Potency as an Oral Vaccine against *Clostridioides difficile*. Vaccines.

[30] Matchett W.E., Anguiano-Zarate S., Malewana G.B.R., Mudrick H., Weldy M., Evert C., Khoruts A., Sadowsky M., Barry M.A. (2020). A Replicating Single-Cycle Adenovirus Vaccine Effective against *Clostridium difficile*. Vaccines.

[31] Senoh M., Iwaki M., Yamamoto A., Kato H., Fukuda T., Shibayama K. (2018). Development of vaccine for *Clostridium difficile* infection using membrane fraction of nontoxigenic *Clostridium difficile*. Microb. Pathog..

[32] Tian J.-H., Glenn G., Flyer D., Zhou B., Liu Y., Sullivan E., Wu H., Cummings J.F., Elllingsworth L., Smith G. (2017). *Clostridium difficile* chimeric toxin receptor binding domain vaccine induced protection against different strains in active and passive challenge models. Vaccine.

[33] Zhang B.-Z., Cai J., Yu B., Hua Y., Lau C.C., Kao R.Y.-T.T., Sze K.-H., Yuen K.-Y., Huang J.-D. (2016). A DNA vaccine targeting TcdA and TcdB induces protective immunity against *Clostridium difficile*. BMC Infect. Dis..

[34] Hensgens M.P.M., Goorhuis A., Dekkers O.M., Kuijper E.J. (2012). Time interval of increased risk for *Clostridium difficile* infection after exposure to antibiotics. J. Antimicrob. Chemother..

[35] Denève C., Janoir C., Poilane I., Fantinato C., Collignon A. (2009). New trends in *Clostridium difficile* virulence and pathogenesis. Int. J. Antimicrob. Agents.

[36] Di Bella S., Ascenzi P., Siarakas S., Petrosillo N., di Masi A. (2016). *Clostridium difficile* Toxins A and B: Insights into Pathogenic Properties and Extraintestinal Effects. Toxins.

[37] Czepiel J., Dróżdż M., Pituch H., Kuijper E.J., Perucki W., Mielimonka A., Goldman S., Wultańska D., Garlicki A., Biesiada G. (2019). *Clostridium difficile* infection: Review. Eur. J. Clin. Microbiol. Infect. Dis..

[38] McDonald L.C., Gerding D.N., Johnson S., Bakken J.S., Carroll K.C., Coffin S.E., Dubberke E.R., Garey K.W., Gould C.V., Kelly C. (2018). Clinical Practice Guidelines for *Clostridium difficile* Infection in Adults and Children: 2017 Update by the Infectious Diseases Society of America (IDSA) and Society for Healthcare Epidemiology of America (SHEA). Clin. Infect. Dis..

[39] Sambol S.P., Tang J.K., Merrigan M.M., Johnson S., Gerding D.N. (2001). Infection of hamsters with epidemiologically important strains of *Clostridium difficile*. J. Infect. Dis..

[40] de Bruyn G., Gordon D.L., Steiner T., Tambyah P., Cosgrove C., Martens M., Bassily E., Chan E.-S., Patel D., Chen J. (2021). Safety, immunogenicity, and efficacy of a *Clostridioides difficile* toxoid vaccine candidate: A phase 3 multicentre, observer-blind, randomised, controlled trial. Lancet Infect. Dis..

[41] Pfizer.com (2022). Phase 3 CLOVER Trial for Pfizer’s Investigational Clostridioides difficile Vaccine Indicates Strong Potential Effect in Reducing Duration and Severity of Disease Based on Secondary Endpoints. https://www.pfizer.com/news/press-release/press-release-detail/phase-3-clover-trial-pfizers-investigational-clostridioides.

[42] Ashford M., Fell J. (1994). Targeting drugs to the colon: Delivery systems for oral administration. J. Drug Target.

[43] Hardy J.G., Healey J.N.C., Reynolds J.R. (1987). Evaluation of an enteric-coated delayed-release 5-aminosalicylic acid tablet in patients with inflammatory bowel disease. Aliment. Pharmacol. Ther..

[44] Wilding I.R., Hardy J.G., Sparrow R.A., Davis S.S., Daly P.B., English J.R. (1992). In vivo evaluation of enteric-coated naproxen tablets using gamma scintigraphy. Pharm. Res..

[45] Kenyon C.J., Cole E.T., Wilding I.R. (1994). The effect of food on the in vivo behavioiur of enteric coated starch capsules. Int. J. Pharm..

[46] Amicizia D., Arata L., Zangrillo F., Panatto D., Gasparini R. (2017). Overview of the impact of Typhoid and Paratyphoid fever. Utility of Ty21a vaccine (Vivotif^®^). J. Prev. Med. Hyg..

[47] Murakami T. (2017). Absorption sites of orally administered drugs in the small intestine. Expert. Opin. Drug Discov..

[48] Heading R.C., Nimmo J., Prescott L.F., Tothill P. (1973). The dependence of paracetamol absorption on the rate of gastric emptying. Br. J. Pharmacol..

[49] Saphier S., Rosner A., Brandeis R., Karton Y. (2010). Gastro intestinal tracking and gastric emptying of solid dosage forms in rats using X-ray imaging. Int. J. Pharm..

[50] Kovacs-Simon A., Leuzzi R., Kasendra M., Minton N., Titball R.W., Michell S.L. (2014). Lipoprotein CD0873 is a novel adhesin of *Clostridium difficile*. J. Infect. Dis..

[51] Staelens D., Liang S., Appeltans B., Van de Wouwer M., Van den Mooter G., Van Assche G., Himmelreich U., Velde G.V. (2016). Visualization of delayed release of compounds from pH-sensitive capsules in vitro and in vivo in a hamster model. Contrast Media Mol. Imaging.

[52] Jacoby H.I. (2017). Gastric Emptying. Biomedical Sciences.

[53] Hunter E., Fell J.T., Calvert R.T., Sharma H. (1980). “In vivo” disintegration of hard gelatin capsules in fasting and non-fasting subjects. Int. J. Pharm..

[54] Yamamoto M., Pascual D.W., Kiyono H. (2012). M cell-targeted mucosal vaccine strategies. Curr. Top. Microbiol. Immunol..

[55] Dukoral Suspension and Effervescent Powder for Oral Suspension, Cholera Vaccine (Inactivated, Oral). https://www.medicines.org.uk/emc/product/5087/smpc#gref.

